# Transient Antenatal Bartter’s Syndrome: A Case Report

**DOI:** 10.3389/fped.2018.00051

**Published:** 2018-03-12

**Authors:** Michelle Meyer, Margarita Berrios, Christina Lo

**Affiliations:** ^1^UCSF Benioff Children’s Hospital Oakland, Oakland, CA, United States

**Keywords:** transient antenatal Bartter’s syndrome, melanoma-associated antigen D mutation, polyhydramnios, polyuria, prematurity

## Abstract

Antenatal Bartter’s syndrome is a rare inherited disorder characterized by fetal polyhydramnios and polyuria that is usually detected between 24 and 30 weeks of gestation. However, a rare, severe, but transient form of antenatal Bartter’s syndrome due to an x-linked melanoma-associated antigen D2 (MAGED2) mutation has recently been described. This transient type results in the earlier onset of severe polyhydramnios and preterm birth, but spontaneously resolves postnatally. Here, we present a case of a 29-week gestation male born to a mother with severe polyhydramnios, who was subsequently found to have a novel mutation for MAGED2 not previously reported. This is the first and only case not to be treated with indomethacin, yet still resulted in spontaneous resolution of symptoms. Our case suggests the need for awareness of and testing for this new mutation in cases of severe antenatal polyhydramnios and discusses the perinatal treatment of this condition.

## Introduction

Bartter’s syndrome, originally described by Bartter and colleagues in 1962, is a renal tubular disorder characterized by hypokalemia, hypochloremia, metabolic alkalosis, and hyperreninemia, with normal blood pressure (1). Clinical features of polyuria are due to impaired sodium chloride reabsorption in the medullary thick ascending limb of the loop of Henle, leading to volume depletion and secondary hyperaldosteronism resulting in hypokalemia from increased urinary potassium excretion and hydrogen ion secretion (1, 2). Hundreds of mutations in genes that regulate the synthesis or insertion of transporters have been identified. Bartter’s syndrome has been classified into types I through V based on severity of symptoms and clinical presentation (3–6). Types I and II, which present in the fetal and newborn periods, are severe disorders, resulting in polyhydramnios during pregnancy and premature delivery. Type I is associated with a SLC12A2 mutation, leading to a defect in the Na-K-2Cl symporter (3), while type II is associated with a mutation in ROMK/KCNJ1, leading to a defect in the potassium channel in the thick ascending limb (4, 5). Type III is less severe than types I and II by presenting later in life and is associated with the loss of activity in the chloride channel Kb (CLC-Kb) (6). Type IV presents in the neonatal period and is associated with mutations in the ClC-Ka and ClC-Kb chloride channels, causing severe disease and sensorineural deafness (7). Type V is associated with a gain-of-function mutation in the CaSR (calcium-sensing receptor), resulting in hypocalcemia and hypomagnesemia (8).

For types I, II, and IV that present in the fetal and newborn periods, treatment of the infant requires lifelong fluid and electrolyte supplementation, as well as the use of non-steroidal anti-inflammatory drugs (NSAIDs) to inhibit excessive renal prostaglandin E_2_ formation, although the safety of long-term treatment with NSAID use, especially in preterm infants, is a subject of controversy. Antenatal treatment with NSAIDs has also been used in cases of severe polyhydramnios to decrease maternal morbidity and to prolong gestation (9, 10).

Recently, a new x-linked variant of antenatal Bartter’s syndrome has been identified that causes a transient syndrome related to a mutation in the melanoma-associated antigen D2 (MAGED2) gene (11). MAGED2 mutations affect the expression and function of NKCC2 and NCC (sodium chloride cotransporters), resulting in poor salt reabsorption in the distal renal tubule. The initial presentation of these patients is that of a type I Bartter’s phenotype, but with more severe symptoms, resulting in earlier onset of polyhydramnios and labor, but all symptoms spontaneously disappear in the infants who survive.

We present a case of a preterm male suspected of having antenatal Bartter’s syndrome, who was subsequently found to have a mutation in the MAGED2 gene causing the transient form.

## Presentation, Diagnosis, and Outcome

The patient was an appropriate for gestational age male infant born at 29 weeks gestation to a G2P2 36-year-old mother with diet-controlled gestational diabetes. Of note, the mother’s first child, also a male, was born full term and healthy, without notable complications. This second pregnancy, however, was complicated by severe polyhydramnios diagnosed at 21 weeks of gestation, with an amniotic fluid index of 30.5 cm (normal amniotic fluid index: 5–24 cm). Despite several courses of indomethacin, the amniotic fluid index ultimately increased to 49 cm, requiring amnioreductions on five separate occasions. Fetal MRI, karyotype, and chromosomal microarray were all normal. The baby was delivered by uncomplicated vaginal delivery after premature rupture of membranes. The mother received betamethasone, magnesium sulfate, clindamycin, and azithromycin during labor. After birth, the baby had severe polyuria, with urine outputs often greater than 20 mL/kg/h requiring aggressive fluid, potassium, and sodium replacement. The patient initially received D5W with 100 mEq/L of sodium chloride and 4 mEq/L of potassium chloride to replace his high urine output and electrolyte deficits. He developed hypokalemia with potassium as low as 2.5 mEq/L (normal values: 3.5–5.3 mEq/L), hyponatremia with sodium as low as 133 mEq/L (normal values: 135–145 mEq/L). This was corrected by adjusting his fluid replacement accordingly and monitoring lab values closely. Fluid balance was managed by monitoring the patient’s hourly urine output and replacing its volume 1:1 plus insensible water losses. Long-term central venous access was necessary to perform frequent blood monitoring and to provide high volume fluid replacement to maintain his fluid and electrolyte balance.

Pharmacological treatment was not used in this case due to variability of response to medications such as NSAIDs and due to concern for potential adverse effects, particularly in premature infants. The infant tolerated supportive management and showed signs of improvement.

Renal ultrasound, done on the third day of life, showed mildly echogenic kidneys, with mild bilateral pelviectasis but no hydronephrosis. A follow-up renal ultrasound on the 14th day of life showed improvement. The serum aldosterone and renin activity levels were elevated to 325 ng/dL and 69.22 ng/mL/h, respectively (normal aldosterone levels for 1–12 months of age are 2–70 ng/dL, with normal renin activity level 0.25–5.82 ng/mL/h). The aldosterone to renin ratio was normal at 4.70 ng/dL/ng/(mL*h) [normal ratio is <20 ng/dL/ng/(mL*h)]. Urine prostaglandins were also elevated at 32 ng/L, which equals approximately 16 ng/24 h (normal < 8 ng/24 h). This constellation of laboratory findings supported a diagnosis of Bartter’s syndrome. Blood was sent to Fulgent Genetics for Next Generation Sequencing, with discovery of a novel hemizygous mutational variant in the MAGED2 gene. Over the course of the first month of life, the patient’s urine output steadily decreased to 2–5 mL/kg/h. As the volume of the patient’s IV replacement fluid decreased, electrolytes were removed and replaced orally. Subsequently, his need for potassium and sodium oral replacement decreased as well. He was discharged at 2 months of age, tolerating breastfeeding plus supplemental NeoSure formula without the need for any electrolyte replacement. At his follow-up visit in the nephrology clinic 29 days after discharge, his serum electrolytes were normal, and he was feeding well with appropriate weight gain.

## Discussion

Although cases of a transient antenatal form of Bartter’s syndrome were reported in the 1990s (12), the entity was poorly characterized until 2016 when Laghmani et al. reported a mutation in MAGED2 in each of 13 male infants born to 7 different families who had transient antenatal Bartter’s syndrome. In their case series, polyhydramnios was recognized at 19–20 weeks of gestation and was diagnosed as severe, with amniotic fluid indices >35 cm. All infants were male, and all were born preterm (median gestational age was 28 weeks, with a range of 22–34 weeks), with seven infants born extremely premature (at less than 28 weeks of gestation). The onset of polyhydramnios and labor began several weeks earlier than previously documented in known types of Bartter’s syndrome. Polyuria, hyponatremia, hypokalemia, hyperaldosteronism, and hypercalciuria were initially present. However, unlike the known types of Bartter’s syndrome, symptoms in these infants lasted from 3 days to 6 weeks, resolving at 30–33 weeks postnatal age. Of note, nephrocalcinosis was noted in six patients and persisted in four patients. Nine patients survived the perinatal period, all of whom were treated with indomethacin for 1–9 years. Whole-exome sequencing identified a MAGED2 mutation in each of these infants who had transient antenatal Bartter’s syndrome. MAGED2 mutations affect the expression and function of NKCC2 and NCC (sodium chloride cotransporters), resulting in poor salt reabsorption in the distal renal tubule. The authors speculated that identification of loss-of-function mutations in MAGED2 in the mothers of male fetuses with acute, early, and severe polyhydramnios may result in the avoidance of unnecessary diagnostic measures in pregnant women and of potentially harmful treatment of preterm babies with indomethacin.

The authors of this case series stated that they did not have an explanation for this transient phenotype. They hypothesized that perhaps an increase in the sensitivity of adenylate cyclase activity to vasopressin, a phenomenon that has been demonstrated during renal development in several species, allows the expression of NKCC2 and NCC independent of MAGED2 after a certain stage in renal development. This would mean that the MAGED2 mutation would no longer be clinically significant after a certain time point in development. MAGED2, in addition, may help to protect NKCC2 and NCC from hypoxia-induced endoplasmic reticulum-associated degradation that occurs antenatally. Postnatally, the renal blood supply is five times its antenatal values, resulting in improved tissue oxygenation. Thus, the protective function of MAGED2 against hypoxia-induced degradation of NKCC2 and NCC may be oxygen dependent and time dependent, becoming less clinically relevant when renal hypoxia resolves postnatally. Future research would need to be conducted to test these hypotheses.

This is the first case report of transient antenatal Bartter’s syndrome due to the MAGED2 mutation after the genetic defect was first described as a distinct entity (9). This is also the first case not to be treated with indomethacin. Our patient survived with resolution of symptoms by 1 month of age and 33 weeks postmenstrual age. He was discharged at 2 months of age on full oral feeds with breast milk and standard formula and no need for pharmacological therapies. He continued to do well after discharge with normal growth, normal electrolytes, and no evidence of nephrocalcinosis. A repeat renal ultrasound at 3.5 months of age was normal.

Our case report, combined with the case reports of 13 male children with the same diagnosis (9), highlights the need for awareness of this syndrome that may be underdiagnosed and underreported, given its recent discovery. This novel MAGED2 variant has an excellent prognosis, with spontaneous resolution of symptoms in early infancy without the need for lifelong medication or electrolyte replacement. *In utero* genetic testing would allow appropriate counseling and avoid unnecessary maternal diagnostic procedures. This case also highlights that this condition can be successfully treated with close monitoring and fluid and electrolyte replacement, avoiding the potential harmful effects of indomethacin. This diagnosis is important for clinical management and parental counseling regarding overall patient prognosis.

## Ethics Statement

Since this is a case report, no protocol or ethics committee was utilized for this report. Any identifiable information has been removed from the manuscript.

## Author Contributions

MM and MB assumed clinical duties of this patient while he was hospitalized and drafted the initial manuscript. MM, MB, and CL all reviewed and revised the manuscript. All authors approved the final case report as submitted and agree to be accountable for all aspects of the work.

## Conflict of Interest Statement

The authors declare that the research was conducted in the absence of any commercial or financial relationships that could be construed as a potential conflict of interest.
